# Application of physiologically based pharmacokinetic modeling for sertraline dosing recommendations in pregnancy

**DOI:** 10.1038/s41540-020-00157-3

**Published:** 2020-11-06

**Authors:** Blessy George, Annie Lumen, Christine Nguyen, Barbara Wesley, Jian Wang, Julie Beitz, Victor Crentsil

**Affiliations:** 1 Center for Drug Evaluation and Research, U.S. FDA, Silver Spring, MD USA; 2 Oak Ridge Institute for Science and Education, Oak Ridge, TN USA; 3 National Center for Toxicological Research, U.S. FDA, Jefferson, AR USA

**Keywords:** Software, Software

## Abstract

Pregnancy is a period of significant change that impacts physiological and metabolic status leading to alterations in the disposition of drugs. Uncertainty in drug dosing in pregnancy can lead to suboptimal therapy, which can contribute to disease exacerbation. A few studies show there are increased dosing requirements for antidepressants in late pregnancy; however, the quantitative data to guide dose adjustments are sparse. We aimed to develop a physiologically based pharmacokinetic (PBPK) model that allows gestational-age dependent prediction of sertraline dosing in pregnancy. A minimal physiological model with defined gut, liver, plasma, and lumped placental-fetal compartments was constructed using the ordinary differential equation solver package, ‘*mrgsolve*’, in R. We extracted data from the literature to parameterize the model, including sertraline physicochemical properties, in vitro metabolism studies, disposition in nonpregnant women, and physiological changes during pregnancy. The model predicted the pharmacokinetic parameters from a clinical study with eight subjects for the second trimester and six subjects for the third trimester. Based on the model, gestational-dependent changes in physiology and metabolism account for increased clearance of sertraline (up to 143% at 40 weeks gestational age), potentially leading to under-dosing of pregnant women when nonpregnancy doses are used. The PBPK model was converted to a prototype web-based interactive dosing tool to demonstrate how the output of a PBPK model may translate into optimal sertraline dosing in pregnancy. Quantitative prediction of drug exposure using PBPK modeling in pregnancy will support clinically appropriate dosing and increase the therapeutic benefit for pregnant women.

## Introduction

Studies have indicated that 64% of pregnant women take at least one medication for the treatment of serious clinical conditions for which cessation of medication in pregnancy is inappropriate^1^. Uncertainty in drug dosing in pregnancy can lead to suboptimal therapy, which can contribute to disease exacerbation during pregnancy. Potential ethical, scientific or legal matters constraint enrollment of pregnant women in clinical trials. In the past five years, only one drug has been approved by the US Food and Drug Administration (FDA) for pregnancy-related indications^2^. As a result, drugs are often prescribed without the necessary clinical knowledge about dose, pharmacokinetics (PK), and safety or efficacy in pregnant women. Without PK data to guide proper dosing, prescribers may use sub-therapeutic doses for pregnant women.

The disposition of drugs can be significantly altered in pregnancy. During pregnancy, a multitude of time-varying physiological and metabolic changes, thought to be regulated by pregnancy-related hormones^3,4^, occur. These changes have a direct effect on drug absorption, distribution, metabolism, and elimination (ADME). The extent of absorption may be diminished in pregnancy due to changes in enzymes or transporters (typically residing in the gut or liver)^5–7^. Changes in drug distribution can occur due to pregnancy-related body weight and fat mass gain, plasma volume expansion, and decrease in plasma protein. Increased hepatic and renal blood flow, glomerular filtration rate and secretion, and changes in hepatic intrinsic clearance can impact total drug clearance during pregnancy. Hepatic intrinsic clearance describes the contribution of hepatic enzymes (mainly cytochrome P450 or CYPs) and transporters to the drug removal process. Drugs may be metabolized by multiple CYP enzymes whose activity may change in opposing directions during pregnancy. For example, CYP3A4, CYP2D6, and CYP2C9 activities are increased during pregnancy, while CYP1A2 and CYP2C19 activities are decreased during pregnancy^8^. Understanding the impact of these pregnancy-related changes on the PK of the drug can be challenging.

Physiologically based pharmacokinetic (PBPK) modeling and simulation combine the knowledge of drug characteristics and physiology of the organism to develop a mechanistic understanding that facilitates the prediction of drug exposure or effects. PBPK models predict target-site specific drug exposure by mapping the complicated mechanistic course of the drug to physiologically realistic compartments using differential equations^3^. PBPK modeling and simulation can account for the physiological changes that occur in pregnancy to predict PK alterations at each stage of pregnancy, making PBPK a potential alternative or a complement to drug trials in pregnancy. PBPK modeling is especially useful in pregnancy since it can integrate time-varying physiologic parameters relevant to drug PK processes, such as changes in maternal weight, organ volumes/blood flows, cardiac output, drug-metabolizing enzyme activities, and glomerular filtration rates, with drug-specific parameters into a quantitative model for prediction beyond the domains of observation. The challenges in obtaining clinical data in special populations such as pregnant women have incentivized increased use of PBPK models for supporting dosing recommendations in drug labels and regulatory decision making^9^. Researchers have demonstrated successful prediction of the disposition of various drugs such as nifedipine, midazolam, and indinavir in the third trimester of pregnancy using PBPK modeling^10,11^.

Major depressive disorder (MDD) is a common but undertreated disorder in pregnancy. Ten to twenty percent of women experience depressive disorders during pregnancy and postpartum^12^. Women with past histories of psychiatric disorders are at a heightened risk of recurrence during pregnancy^13^. Untreated depression during pregnancy can lead to impaired self-care, failure to follow prenatal guidelines, suicidality, and impulsivity that can endanger the health of mother and child^14^. The risk versus benefit analysis favors the treatment of depression during pregnancy; nonetheless, there are no established dosing guidelines for treatment of depression during pregnancy. The few studies that have been conducted report there is a need to increase antidepressant doses as pregnancy advances^15^. However, increasing the dose arbitrarily can result in surpassing the optimal dose for maximizing maternal therapeutic benefit and minimizing fetal risk.

Sertraline is a selective serotonin reuptake inhibitor (SSRI) and is one of the first-line agents for treating depression. It is slowly absorbed after oral administration, peaking after 6 to 8 h in the plasma^16^. Sertraline exhibits linear kinetics with an elimination half-life of 32 h and is dosed once daily. Sertraline is also highly protein-bound (98.5%)^17^. Metabolism of sertraline is thought to be mediated by five or more different CYP enzymes including CYP3A4, CYP2B6, CYP2C9, CYP2C19, and CYP2D6 which convert sertraline to an inactive metabolite, desmethylsertraline, in the liver^18,19^. Renal clearance of sertraline was found to be negligible^16^. We used sertraline as a case-study drug to develop a pregnancy PBPK model and a prototype for an interactive dosing tool that will support dose optimization in pregnancy.

## Results

### Nonpregnancy PBPK model calibration

**Fig. 1:**
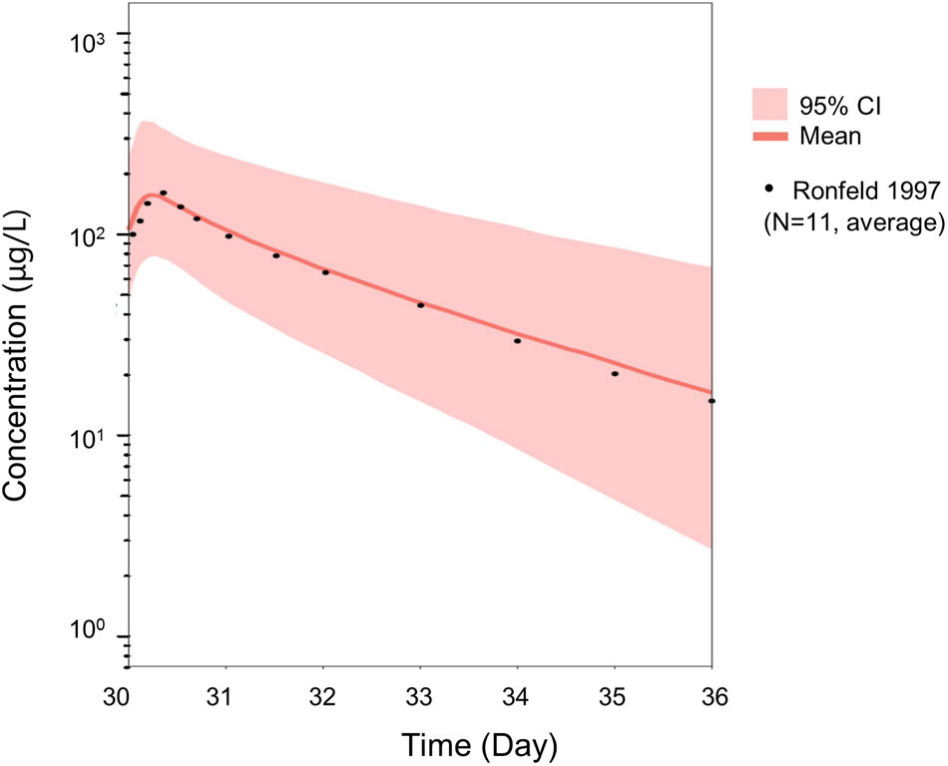
Model prediction for steady-state sertraline plasma concentrations in nonpregnancy. Black dots represent mean total plasma concentration in nonpregnant females (*N* = 11) ages 20–45 years receiving sertraline 200 mg oral tablets daily for 30 days following the last dose^17^. Monte Carlo simulations with a daily dose of 200 mg for 30 days were run for 1000 iterations. The predicted mean is depicted by the red line. The 95% prediction interval (2.5th–97.5th percentile range of a virtual population [*N* = 1000]) is depicted in the red area.

The nonpregnancy PBPK population model, following calibration, was simulated with the conditions of the Ronfeld study (200 mg oral tablets sertraline daily for 30 days) and compared to the observed sertraline plasma concentration (*N* = 11)^17^. The predicted plasma concentration versus time profile matched the observed profile of sertraline well (Fig. 1). The observed mean and the model prediction for the 50th percentile for several of the PK parameters in nonpregnancy were comparable (Table 1). The 50th percentile prediction for *T*_max_ and *T*_1/2_ were slightly underpredicted, however, the prediction ranges overlapped with the observed parameter variance for *T*_max_. Monte Carlo simulations (*N* = 1000) were used to derive the 95% prediction interval around the mean plasma concentrations. The standard deviations for the observed PK parameters were within the 95% prediction interval.

**Table 1:** Pharmacokinetic parameters observed versus predicted in nonpregnancy.

Parameter	Observed^a^	Predicted^b,c^
	(Mean ± SD)	50th (2.5–97.5th percentile)
AUC_24_ (μg/L•h)	3063 ± 1413	3092 (1387–7879)
*C*_max_ (μg/L)	166 ± 65	159 (71–433)
*T*_max_ (h)	6.7 ± 1.8	5.0 (3.0–6.0)
*T*_1/2_ (h)	32.1	26.2 (24.0–28.0)

^a^Ronfeld et al.^17^ (*N* = 11).

*SD* standard deviation, *AUC*_*24*_ area-under-the-curve from time 0 to 24 hours, *C*_*max*_ maximum plasma concentration, *T*_*max*_ time to maximum plasma concentration, *T*_*1/2*_ half-life.

^b^Pharmacokinetic parameters calculated by Non-Compartmental Analysis from model predicted sertraline plasma concentrations in nonpregnancy.

^c^Monte Carlo Simulation (*N* = 1000).

### Pregnancy PBPK model evaluation

**Fig. 2:**
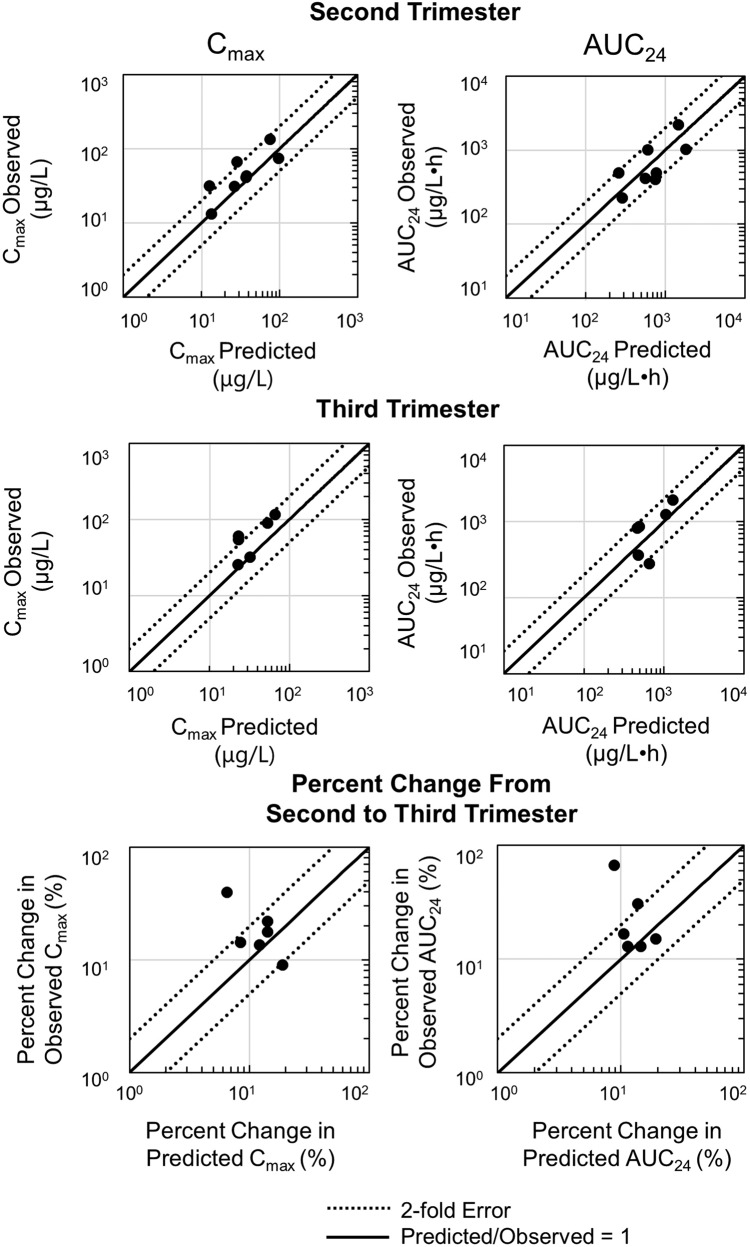
Pregnancy model evaluation in second and third trimester of pregnancy. The predicted versus observed graphs for each pharmacokinetic (PK) parameter (maximum plasma concentration [*C*_max_] and 24-h drug exposure [AUC_24_]) is given for second (*N* = 8) and third trimester (*N* = 6). In addition, the predicted versus observed graph for percent change from second to third trimester is also shown (*N* = 6). The solid line represents the unity line where the predicted to observed ratio is 1. The dotted lines represent the twofold error. The black dots represent individual patient data.

Pregnancy model simulations for each subject during the second and third trimesters were compared with observed maximum plasma concentration (*C*_max_) and 24-h drug exposure (AUC_24_) in the second and third trimesters of pregnancy (Fig. 2). The accuracy as indicated by the average fold error (AFE) value was calculated for *C*_max_ and AUC_24_ for both the second and third trimester. An AFE greater than one suggests bias towards overprediction whereas an AFE less than one suggests a bias towards underprediction. The AFE value for *C*_max_ was 0.6 for the second trimester and 0.5 for the third trimester. The AFE value for AUC_24_ was 0.9 for the second trimester and 0.7 for the third trimester. The precision as indicated by the average absolute fold error (AAFE) value for both C_max_ and AUC_24_ ranged from 1.5–1.7. For *C*_max_, 6 out of 8 subjects and 4 out of 6 subjects were within a twofold error of the observed for the second trimester and third trimester, respectively. For AUC_24_, 8 out of 8 subjects and 6 out of 6 subjects were within a twofold error of the observed for the second trimester and third trimester, respectively. Furthermore, in the observed data, the average *C*_max_ decreased by 15% (range: 9–21%) from second to the third trimester and the average AUC_24_ decreased by 17% (range: 12–30%) from second to the third trimester. This data aligns with the predicted model results as shown in Table 2. Notably, one patient was excluded from the above calculation. The PK parameters of this patient deviated from the others by showing an increase in *C*_max_ and AUC_24_ from second to the third trimester, while the other five patients showed a decrease. Therefore, it is likely that this patient is an outlier. For 4 out of 6 subjects, the predicted *C*_max_ percent change from second to the third trimester was within a twofold error of the observed. For 5 out of 6 subjects, the predicted AUC_24_ percent change from second to the third trimester was within a twofold error of the observed (Fig. 2).

**Table 2:** Predicted percent change in pharmacokinetic parameters across gestational age compared to nonpregnancy.

Gestational age (weeks)^a^	*C*_max_	AUC_24_	CL/F	*V*_d_
7	−6%	−8%	9%	3%
20	−32%	−35%	53%	8%
34	−53%	−54%	119%	14%

*C*_*max*_ maximum plasma concentration, *AUC*_*24*_ area-under-the-curve from time 0 to 24 h, *CL/F* apparent clearance (clearance/bioavailability), *V*_*d*_ volume of distribution

^a^Percent change in sertraline pharmacokinetic parameters for a representative gestational age for each trimester compared to nonpregnancy across therapeutic dose ranges (50–200 mg).

### PBPK model predicted change in sertraline hepatic metabolism and plasma clearance

**Fig. 3:**
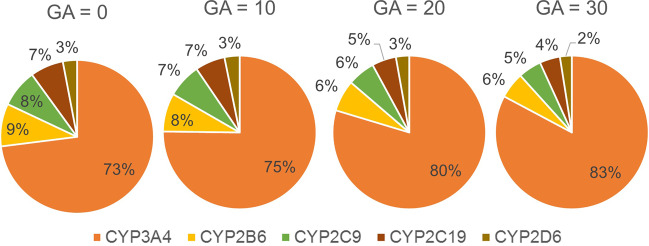
Predicted change in hepatic enzyme contribution to sertraline metabolism across gestation. The nonpregnancy contribution of five cytochrome P450 (CYP) enzymes to sertraline metabolism was calculated by in vitro-in vivo extrapolation^19^. Following incorporation of gestation-dependent increase in CYP activities, the contributions of each individual CYP enzyme out of the total hepatic clearance at various (0, 10, 20, and 30) gestational ages (GA) are demonstrated. Note that the total hepatic clearance increased over the course of pregnancy by 11%, 37%, and 63% during GA 10, 20, and 30 weeks, respectively.

**Fig. 4:**
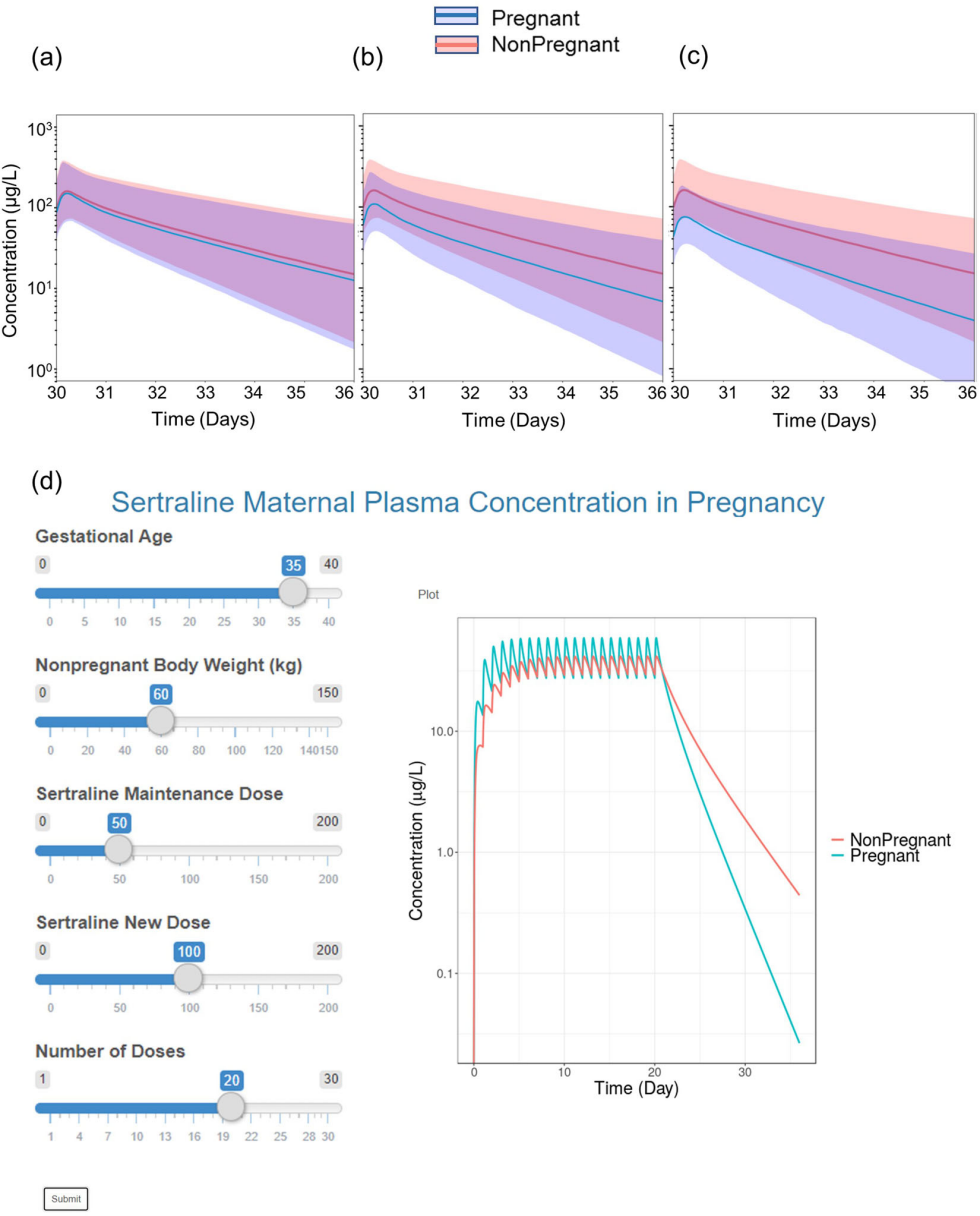
Predicted change in steady-state sertraline plasma concentrations with gestational age and interactive PBPK dosing tool for sertraline. **a**–**c** The predicted sertraline plasma concentration in pregnancy (blue) compared to nonpregnancy (red) is visually depicted. Representative gestational ages (GAs) for each trimester is shown in graphs (**a**–**c**). Lines represent the mean concentration while the colored areas represent the 95% prediction interval (2.5th–97.5th percentile range of a virtual population [*N* = 1000]). **d** A screenshot for the web-based interactive PBPK dosing tool. Users can adjust various parameters including gestational age, body weight, dose and number of doses. Please note that the current version of the tool is a prototype and includes mean plasma concentration versus time profiles for illustrative purposes and does not include estimates of computed population variabilities.

Since the model assumed that sertraline clearance was solely through the liver, we explored the changes in the predicted CYP enzyme contributions to sertraline metabolism (Fig. 3). Based on the IVIVE calculations, in the nonpregnancy model CYP3A4 metabolism constituted 73% of the total metabolism. CYP2B6, CYP2C9, CYP2C19, and CYP2D6 contributed 9%, 8%, 7% and 3%, respectively. Equations for gestational-dependent changes in CYP activity were only available for CYP3A4 and CYP2D6. Following the incorporation of gestational-dependent equations, which capture the increase in CYP3A4 and CYP2D6 activity, total hepatic intrinsic clearance increased by 11%, 37%, and 63% at Gestational Age (GA) 10, 20, and 30 weeks, respectively. Maximally, the model predicted up to a 143% increase in sertraline plasma clearance during the third trimester (GA = 40 weeks) compared to nonpregnancy. A maximal decrease of 59% exposure to sertraline was predicted in the third trimester. The extent of the changes described above was similar across various therapeutic doses (50–200 mg). Change in PK parameters for representative gestational ages for each trimester compared to nonpregnancy is shown in Table 2. Plasma curves for representative gestational ages and nonpregnancy are shown in Fig. 4a–c. Based on our pregnancy model predictions, the sertraline dose in pregnancy needed to maintain equivalent nonpregnancy exposure is estimated to be increased by 9%, 52%, and 117% in trimester 1, 2, and 3, respectively, from the non-pregnancy dose.

### PBPK model web dosing tool

The PBPK model was converted to a prototype web-based interactive dosing tool to facilitate comparison of sertraline dosing in pregnancy versus nonpregnancy (Fig. 4d). Using the user-friendly interface, non-modelers can easily use and apply the PBPK model to predict and compare maternal plasma concentrations of sertraline in various gestational ages of pregnancy and nonpregnancy. Users can also simulate a dose adjustment with commercially available dose strengths of sertraline to attain equivalent nonpregnancy drug exposure.

## Discussion

In the present study, a comprehensive PBPK modeling framework with a user-friendly interface was developed using a drug commonly used to treat depression in pregnant women as an example. PBPK modeling has been a valuable tool for regulatory science and can become a powerful tool in the hands of clinicians. The scarcity of user-friendly tools for modeling has limited the widespread utility of PBPK. Commercial PBPK software programs are well accepted for various PK analysis; however, their complexity may deter non-modelers^20^. The current PBPK framework is based on R programming language, allowing for flexibility and transparency compared to commercial software programs. The web-based dosing tool for the PBPK model can be viewed as a standalone software but also has the flexibility to be updated and revised and will help translate the output of the PBPK model into optimal drug dosing in pregnancy. As a prototype, the routine use of this web-based tool by clinicians at the point of care will be premature and not advisable.

**Fig. 5:**
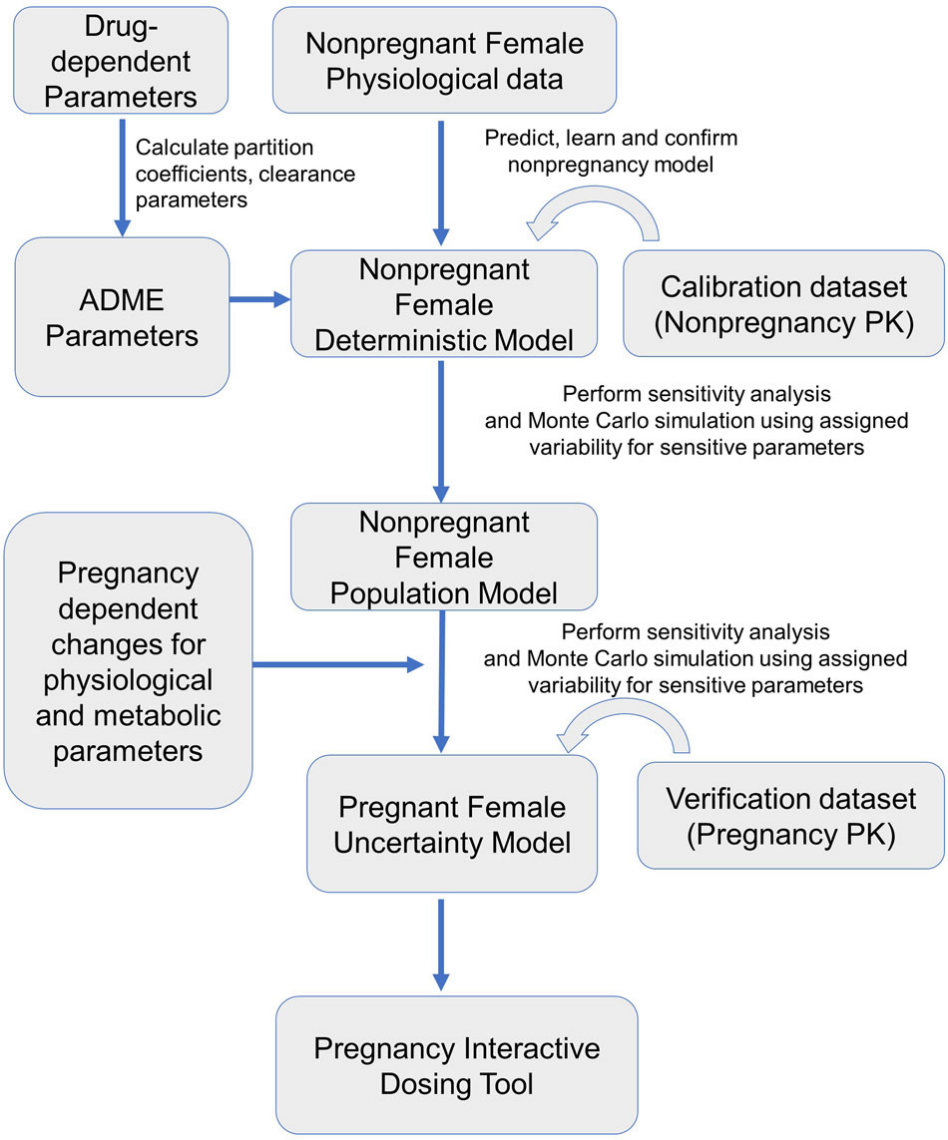
Workflow for pregnancy physiologically based pharmacokinetic model. Workflow for the development of the pregnancy physiologically based pharmacokinetic (PBPK) model. Sertraline physicochemical properties were collected from DrugBank and used to calculate absorption, distribution, metabolism, and excretion (ADME) parameters^29^. Physiological data for nonpregnancy was taken from ICRP Publication 89^28^. A deterministic nonpregnancy PBPK model was established and calibrated with pharmacokinetic data in nonpregnancy from a calibration dataset^17^. Following satisfactory calibration, population prediction was achieved by performing sensitivity analysis and Monte Carlo simulations. The population nonpregnancy model was extended to pregnancy by incorporating physiological changes in pregnancy^4,44,45^. The pregnancy model was simulated to predict pharmacokinetic data for the second and third trimester of pregnancy using a verification dataset^30^. Following verification, an interactive pregnancy dosing tool was created using ‘*Shiny’*.

The PBPK model for sertraline that we established for pregnancy and nonpregnancy highlights the detailed procedure for the comprehensive PBPK modeling framework development (Fig. 5). The PBPK model successfully predicted the observed sertraline exposures in nonpregnancy and pregnancy. The population simulations with the Monte Carlo method illustrate that the variability and uncertainty around the predictions can be modeled. The approach that we followed can be adapted to predict the change in plasma concentrations of other drugs across increasing gestational age, allowing dose adjustments as the pregnancy progresses. Although the model has limitations, it lays a foundation to encourage prediction-based approaches to dosing in pregnancy in a user-friendly and real-time manner.

The initial model structure was based on the physicochemical characteristics and clinical pharmacology of sertraline. Modeling of the parent drug was prioritized due to the metabolites of sertraline being inactive^21^. Although there is a concern for CYP3A4 metabolism inhibition by sertraline and its metabolites, the reported in vitro inhibition constants are much higher than the observed circulating metabolite concentrations^22^. Initially, a first-order rate absorption was used in our model; however, the *C*_max_ and time at the maximum concentration (*T*_max_) were not adequately captured. Following the addition of enterohepatic circulation and a time-dependent absorption rate, the *C*_max_ and *T*_max_ reflected the PK profile of the calibration dataset better. Furthermore, there is evidence for the enterohepatic circulation of sertraline in the literature^23–25^. Sertraline is a class II (low solubility, high permeability) compound based on the Biopharmaceutics Classification System (BCS), which limits absorption from the gut due to solubility^26^. The time-dependent absorption rate constant (*K*_a_) we incorporated into the model will account for the time it takes sertraline to dissolve and be available for absorption^27^. Time-dependent absorption parameters and enterohepatic circulation parameters in our model were estimated to fit the calibration dataset. However, as indicated by the AFE, there is still underprediction of *C*_max_ in our pregnancy PBPK model. This could be due to our assumption, due to lack of observed data, that absorption of sertraline does not change in pregnancy.

Based on the sensitivity analysis (Supplementary Fig. 2), several parameters had major influences on the prediction of sertraline plasma concentrations. The highly influential parameters include fraction of unbound drug (Fu), intrinsic clearance, body weight, volume of slowly and richly perfused tissues, and liver volume. The nonpregnancy sertraline Fu value (mean and standard deviation) was taken from experimental measurements in the Ronfeld study while the nonpregnancy physiological values such as body weight and volume of organs were reference values for average females from ICRP Publication 89^17,28^. Nonpregnancy intrinsic clearance was estimated based on an IVIVE method. There is controversy as to the exact contributions of various CYP450 enzymes to sertraline metabolism. We calculated separate clearance values using two published in vitro studies^18,19^. The clearance value calculated using the Obach et al.^19^ in vitro studies gave the best fit to the calibration PK dataset. In the pregnancy model, the gestational-dependent change in Fu was based on the equation provided in Supplementary Table 4. Fu increased by ~3%, 12%, and 27% during the first, second, and third trimesters, respectively. The change in body weight during pregnancy was determined by the equation provided in Supplementary Table 4. This translated into an increase in sertraline distribution to richly and slowly perfused tissues based on the growth of pregnancy-specific compartments or as body fat, respectively. Total intrinsic clearance increased over the course of pregnancy, based on increases in CYP3A4 and CYP2D6 activity (equations provided in Supplementary Table 4). The increase in sertraline clearance in pregnancy seen in our model is attributed to these multifactorial physiological changes in pregnancy and the complex interplay of these factors.

A limitation of the pregnancy PBPK model was the unavailability of the gestational-age dependent equations for CYP2C19, CYP2B6, and CYP2C9. However, the sensitivity analysis for the pregnancy PBPK model revealed that CYP3A4 had the most impact on the pregnancy model compared to the other CYPs (Supplementary Fig. 2B). To also mitigate this limitation, the hepatic intrinsic clearance parameter was assigned variability and uncertainty during Monte Carlo simulations in our model. If data on other CYPs becomes available in the future, it can be easily incorporated.

Due to the lack of substantial sertraline PK data in pregnant women, the predictive performance evaluation of our model was limited. To date, there are no published sertraline PK studies in pregnant women with richly sampled time points. Furthermore, other covariates such as genetic polymorphisms in CYP enzymes, inherent parameter correlations and co-dependencies that add to PK variability were not evaluated in our study. The minimal PBPK model we developed with the currently available PK and physiological data provide a simplified approach to quantifying sertraline disposition in nonpregnancy that translated well into pregnancy. We foresee that this approach can be generalized to any drug used in pregnancy. Future studies that capture PK data in pregnant and nonpregnant women can be used to further validate our model.

## Methods

Figure 5 depicts the workflow of the present study. In brief, we developed a minimal nonpregnancy model for sertraline, using typical physiological parameters for females^28^ relevant ADME processes^16,19^, and physicochemical data^29^. Once the nonpregnancy PBPK model was adjusted satisfactorily to the calibration dataset (observed clinical PK data in nonpregnancy^17^), we incorporated variability and uncertainty as well as implemented the physiological changes in pregnancy. Finally, the simulated maternal PK profiles were compared with observed clinical PK data in the second and third trimesters for model verification^30^. The model was developed using R software (Version 3.5.0) with the ordinary differential equation (ODE) solver package ‘*mrgsolve*’ (Version 0.8.12)^31,32^. An interactive dosing tool was developed with the ‘*shiny*’ package (Version 1.4.0.2) based on the R model code.

### PBPK model structure

**Fig. 6:**
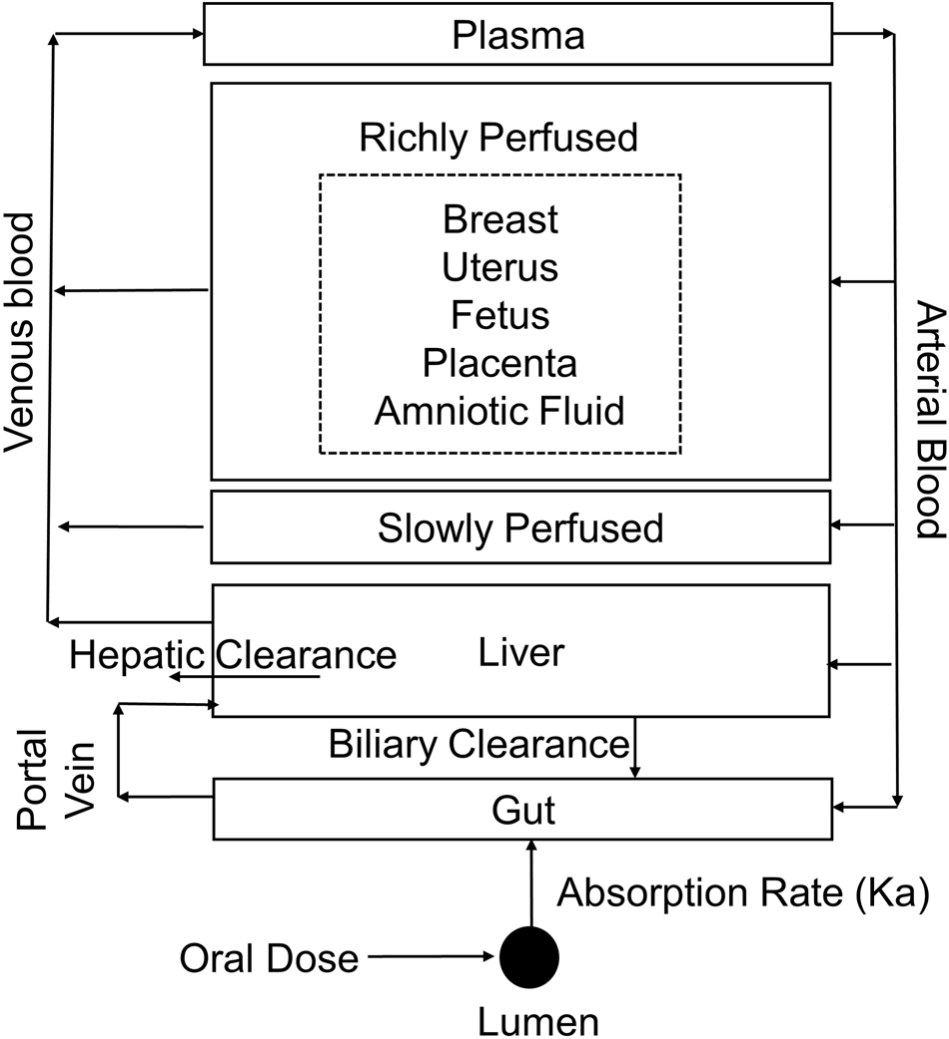
Minimal pregnancy PBPK model structure for sertraline. Physiologically based pharmacokinetic (PBPK) model structure for sertraline after oral exposure. Arrows represent blood flow between compartments. Boxes represent tissue compartments described as flow limited. Pregnancy-related tissues and remaining richly perfused and slowly perfused tissues are represented as lumped compartments. *K*_a_ represents the absorption rate constant for disappearance from lumen and appearance in the gut compartment.

The minimal nonpregnancy PBPK model structure consists of five compartments corresponding to different tissues in the body including the liver, gut, plasma, and the rest of the body divided into lumped slowly and richly perfused tissues connected by circulating blood system (Fig. 6). The corresponding equations are provided in Supplementary Table 5. The choice of compartments was defined by sertraline ADME processes. Each compartment was defined by a tissue volume and blood flow rate. The perfusion-limited model which works well for small molecular weight compounds was applied to the current model. The minimal pregnancy PBPK model included a lumped placental-fetal compartment that was added to the richly perfused tissues compartment (Fig. 6).

### Non-pregnancy PBPK model parameterization

Distribution to various tissues was determined by drug-tissue partition coefficients. Partition coefficients represent the numerical ratio of the drug concentration in tissues and plasma at equilibrium. Partition coefficients were estimated using the tissue composition-based technique described by Poulin and Haddad for highly lipophilic compounds and adjusted to fit the curve of the calibration dataset^33^. The partition coefficients and the physicochemical properties used in the model are provided in Supplementary Tables 1 and 2. The fraction unbound value used was experimentally determined^17^. The log P and pKa values used were obtained from DrugBank^29^.

In our model, we have assumed sertraline plasma clearance is due to 100% metabolism by CYP3A4, CYP2B6, CYP2C9, CYP2C19, and CYP2D6^18,19^. Quantitative estimates of the CYP-specific and total hepatic clearance for sertraline, were derived using in vitro-in vivo extrapolation (IVIVE) (Supplementary Fig. 1). In brief, the in vitro intrinsic clearance in recombinant human P450 enzymes was calculated for each individual CYP enzyme by dividing the reported maximum rate of reaction (*V*_max_) by the substrate concentration (0.5 µM) reported in the Obach study^19^. Then, the intrinsic clearances were multiplied by a recombinant-to-microsomal enzyme activity conversion factor also reported by Obach^19^. We assumed the fu_mic_ or fraction unbound in microsomal systems to be 1, as this value was not reported by Obach. The intrinsic clearances were then scaled to in vivo by multiplying with microsomal protein per gram of liver (MPPGL) which was set at 40 mg/g, as well as mean enzyme abundance in nonpregnant healthy individuals^34^. Finally, the individual intrinsic clearances for the five CYP enzymes were added to get the total in vivo hepatic intrinsic clearance.

### Non-pregnancy PBPK model calibration

The deterministic nonpregnancy PBPK model was adjusted using a calibration dataset which consisted of a single study by Ronfeld et al.^17^ with measured time-plasma concentration data for sertraline in 11 nonpregnant women ages 20–45 years. In the Ronfeld study, sertraline 50 mg tablets were gradually titrated to a maximum of 200 mg/day and administered daily for 30 days. Serial blood samples were collected following the last dose on day 30. The average concentration-time data (*N* = 11) was extracted from the graph provided using WebPlotDigitizer (version 4.2, https://apps.automeris.io/wpd/).

### Sensitivity analysis and Monte Carlo Simulations

A local sensitivity analysis was performed at steady state to investigate the influence of each model input parameter on predicting *C*_max_ and area under the plasma-concentration curve for 24 h (AUC_24_). Model parameters with a 10% increase in the input parameter leading to a 0.5% change in *C*_max_ or AUC_24_ are reported in Supplementary Fig. 2 for a comprehensive view of model sensitivities. Normalized sensitivity coefficients (SC) were determined according to the following equation for each individual condition tested:

1 $${\mathrm{SC}} = \frac{{({\mathrm{MO}}^\prime - {\mathrm{MO}})}}{{({\mathrm{IP}}^\prime - {\mathrm{IP}})}} \times \frac{{{\mathrm{IP}}}}{{{\mathrm{MO}}}},$$

where MO is the initial value of the model output, MO′ is the modified value of the model output resulting from an increase in the input parameter value, IP is the initial input parameter value, and IP′ is the modified parameter values^35^. Sensitivity analysis results for nonpregnancy model is provided in Supplementary Fig. 2A. Sensitivity analysis was also performed for the pregnancy model for a representative gestational age (Supplementary Fig. 2B).

Monte Carlo simulations were employed to estimate the effects of parameter uncertainty and inter-subject variability on model simulations. One thousand iterations were carried out for each Monte Carlo analysis with sensitive model parameters randomly selected from defined distributions as described in Supplementary Table 3. Log-normal distributions of model parameters were assumed for all drug-specific parameters such as partition coefficients, absorption rate constants, elimination rate constants, etc. Physiological parameters, including fractional blood flows and tissue volumes were assumed to be normally distributed, except for body weight and cardiac output which were assumed to be lognormally distributed^36–39^. Probabilistic distributions of model parameter values were derived from experimental data when available^4^. For physiological parameters for which no experimental data were available, coefficients of variation (CVs) were assigned as 20% for partition coefficients and 30% for physiological parameters, absorption, and elimination rate constants based on the default assumptions used in other PBPK models^40–43^. To ensure that randomly selected parameter values were biologically plausible, the 2.5th and 97.5th percentiles of each parameter were calculated as the upper and lower bounds for the truncated sampling distribution and listed in Supplementary Table 3. To preserve mass balance when physiological parameters were randomly chosen based on distributions, adjustment factors were used so that the sum of fractions of tissue volumes or blood flows would equal 1. Due to the low sensitivity of pregnancy-specific compartments (Supplementary Fig. 2B), the dynamic variability in pregnancy-related changes were assumed to be the same for each subject.

### Extrapolation and evaluation of pregnancy PBPK model

Pregnancy-related changes were incorporated as gestational-dependent polynomial equations adapted from several publications^4,44,45^. The equations used in our model are provided in Supplementary Table 4. The pregnancy PBPK model was evaluated with a verification dataset with PK data not applied to the model calibration. The verification dataset consisted of a single published study in which PK data (*C*_max_ and AUC_24_) for second and third trimesters were collected from 8 and 6 subjects, respectively^30^. Various oral doses (25–200 mg) of sertraline were taken daily by pregnant women for at least 2 weeks. Serial blood samples were collected during the second trimester (22–26 weeks) and third trimester (30–34 weeks). Monte Carlo simulations were conducted by entering each patient’s current body weight calculated back to pre-pregnancy body weight based on the time-varying equation for body weight gain in pregnancy^4^, the gestational age range for each trimester visit, and the corresponding reported oral dose. PK parameters were calculated by Non-Compartmental Analysis using the ‘*PKPDmisc*’ package (Version 2.1.1) in R for the median predicted plasma curve after 1000 iterations.

### Pregnancy PBPK model predictive performance

We compared the *C*_max_ and AUC_24_ predictions based on body weight and gestational age to the observed *C*_max_ and AUC_24_ values for each subject. The bias and precision of the PK parameters were assessed through calculation of AFE and AAFE using the following equations^46^:

2 $${\mathrm{AFE}} = 10^{1/N{\Sigma}{\mathrm{log}}({\mathrm{Predicted/Observed}})},$$

3 $${\mathrm{AAFE}} = 10^{1/N{\Sigma}\left| {{\mathrm{log}}({\mathrm{Predicted/Observed}})} \right|}.$$

### PBPK model web dosing tool

The PBPK model was converted to a prototype web-based interactive dosing tool to facilitate comparison of sertraline dosing in pregnancy versus nonpregnancy by non-modelers. The interface was constructed with the ‘*Shiny*’ package. A screenshot of the tool is shown in Fig. 4d.

## Disclaimer

This article reflects the views of the authors and should not be construed to represent the views or policies of the U.S. Food and Drug Administration.

### Reporting summary

Further information on experimental design is available in the Nature Research Reporting Summary linked to this paper.

## Supplementary information

Supplementary Material

Reporting Summary Checklist

## Data Availability

All data generated or analyzed during this study are included in this published article (and its Supplementary Files).

## Code availability

The codes supporting the current study are available from the corresponding author upon request.
