# Supplementary material for: Application of physiologically based pharmacokinetic modeling for sertraline dosing recommendations in pregnancy
Source: NPJ Syst Biol Appl. 2020 Nov 6;6:36. doi: 10.1038/s41540-020-00157-3 (PMC7648747; doi:10.1038/s41540-020-00157-3)
Supplement: Supplementary file 1 — Supplementary Material [file 41540_2020_157_MOESM1_ESM.pdf]

Recombinant Human CYP450 Enzymes

Intrinsic Clearance ($\mu L\ min^{-1}\ pmol^{-1}$)	
CYP3A4	0.12
CYP2B6	0.32
CYP2C9	0.06
CYP2C19	0.9
CYP2D6	0.8

→

Recombinant to Microsomal Enzyme Activity conversion

Relative Activity Factor	
CYP3A4	48
CYP2B6	32
CYP2C9	23
CYP2C19	7
CYP2D6	6

→

MPPGL: Microsomal Protein Per Gram of Liver 4×10^4 mg per kg (adult human liver)

→

Enzyme Abundance Non-Pregnancy ($pmol\ mg^{-1}$)	
CYP3A4	155
CYP2B6	11
CYP2C9	73
CYP2C19	14
CYP2D6	8

→

Unit conversion uL per min to L per hr

→

Intrinsic Clearance $L\ hr^{-1}\ kg^{-1}$ (% of Total)	
CYP3A4	2143 (73)
CYP2B6	266 (9)
CYP2C9	242 (8)
CYP2C19	212 (7)
CYP2D6	92 (3)

Supplementary Figure 1. In-Vitro-In-Vivo-Extrapolation (IVIVE) for Sertraline Metabolism and Clearance. The hepatic intrinsic clearance was calculated based on IVIVE method. Each individual CYP450 clearance rate was calculated from *in vitro* experiments conducted by Obach et al. in recombinant human cytochrome P450 (CYP450) enzymes measuring the conversion of 0.5 µM sertraline to N-desmethylsertraline[1]. The individual CYP450 clearance rate was scaled to *in vivo* using several conversion factors as shown above. The sum of the individual CYP450 intrinsic clearances was used to calculate total hepatic clearance in the nonpregnant PBPK model.

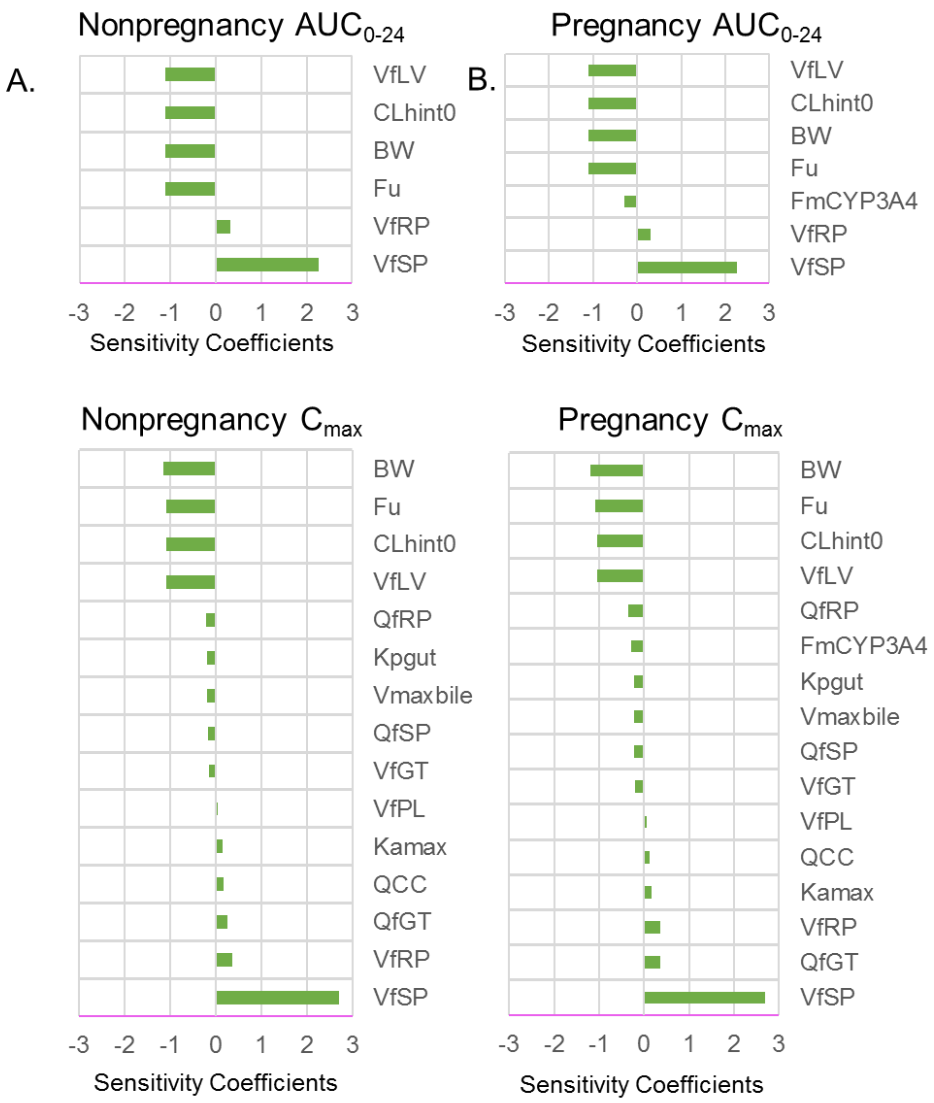

Supplementary Figure 2. Sensitivity Analysis for Nonpregnancy and Pregnancy PBPK Model. Analysis of the sensitivity of the model to a 10% increase in each parameter as reflected by the model predicted changes in 24-hour drug exposure (AUC_{24}) or maximum plasma concentration (C_{max}) is shown for nonpregnancy **(A)** and a representative gestational age (GA=22) in pregnancy **(B)**. Calculated changes in AUC_{24} and C_{max} for the sensitive input parameters are shown in the units of sensitivity coefficients (SCs). Only parameters with SC > 0.05 or < -0.05 are displayed.

BW: Body Weight, Clhint0: Intrinsic Clearance, FmCYP3A4: Fraction metabolized by Cytochrome P450 3A4, Fu: Fraction of unbound Drug, Kamax: Maximum Rate of Absorption, Kpgut: Gut Partition Coefficient, QCC: Cardiac Output Scaling Factor, QfGT: Fraction of Blood Flow to Gut, QfRP: Fraction of Blood Flow to Richly Perfused Tissue, QfSP: Fraction of Blood Flow to Slowly Perfused Tissue, VfGT: Fraction of Gut Weight, VfLV: Fraction of Liver Weight, VfPL: Fraction of Plasma Weight, VfRP: Fraction of Richly Perfused Tissue Weight, VfSP: Fraction of Slowly Perfused Tissue Weight, Vmaxbile: Maximum Rate of Bile Emptying.

Supplementary Table 1. Physicochemical Properties of Sertraline used for Model Development

Parameter	Value	Reference
Plasma Fraction Unbound (Fu)	0.015	2
Partition coefficient ($Log\ P_{octanol:water}$)	5.15	3
Partition coefficient ($Log\ P_{vegetable\ oil:water}$)	5.25	3
Dissociation constant (pKa)	9.5	3

Supplementary Table 2. Partition Coefficients of Sertraline used for Model Development

Partition Coefficient	Value	Reference
Gut	1.7	4
Liver	2.18	4
Slowly Perfused Tissues	25	Fitted
Richly Perfused Tissues	2	Fitted

Supplementary Table 3. Values and Distributions of Parameters used in Monte Carlo Simulation for the Nonpregnancy PBPK Model of Sertraline

Parameter	Abbreviation	Distribution	Mean	SD	CV	Lower Bound	Upper Bound	Reference
Body weight	BW	Lognormal	62.1	9.32	0.15	45.8	82.3	5,2
Fraction unbound	Fu	Lognormal	0.015	1.35×10^{-3}	0.09	0.0125	0.0178	2
Intrinsic Clearance	CLhint0	Lognormal	2958.7	739.7	0.25	1771.5	4650.7	IVIVE
Fraction of Liver Weight	VfLV	Truncated normal	0.024	0.0072	0.3	0.0099	0.038	6
Fraction of Slowly Perfused Tissue Weight	VfSP	Truncated normal	0.674	0.2022	0.3	0.278	1.07	7
Fraction of Richly Perfused Tissue Weight	VfRP	Truncated normal	0.221	0.0663	0.3	0.091	0.351	7
Fraction of Blood Flow to Gut	QfGT	Truncated normal	0.17	0.051	0.3	0.07	0.27	6
Gut Partition Coefficient	Kpgut	Lognormal	1.7	0.34	0.2	1.13	2.46	Fitted
Cardiac Output Scaling Factor	QCC	Lognormal	14.9	1.341	0.09	12.4	17.7	6
Fraction of Gut Weight	VfGT	Truncated normal	0.024	0.0072	0.3	0.0099	0.038	6
Rate of Maximum Absorption	Kamax	Lognormal	0.5	0.15	0.3	0.269	0.851	Fitted
Fraction of Blood Flow to Slowly Perfused Tissues	QfSP	Truncated normal	0.211	0.0633	0.3	0.0869	0.335	7
Fraction of Blood Flow to Richly Perfused Tissues	QfRP	Truncated normal	0.519	0.1557	0.3	0.214	0.824	7
Fraction of Plasma Weight	VfPL	Truncated normal	0.04	0.0064	0.16	0.0274	0.0525	6
SD: Standard Deviation, CV: Coefficient of Variation								

Supplementary Table 4. Equations Used for Gestational-Dependent Changes in Pregnancy

Equation	Reference
$CO = 301 + 5.916GA - 0.088GA^2$	5
$Qgut = (QGT - (0.1125GA)/100)*CO$	8
$Qliver = (QLV - (0.0625GA)/100)*CO$	8
$Quterus = 1.71 + 0.2068GA + 0.0841GA^2 - 0.0015GA^3$	5
$BW = 61.1 + 0.2409GA + 0.0038GA^2$	5
$Vplasma = 2.5 - 0.0223GA + 0.0042GA^2 - 0.00007GA^3$	5
$Vbreast = 985 + 14.244GA - 0.1869GA^2$	5
$Vuterus = 80 + 8.29321GA + 0.3546GA^2$	5
$Vfetus = (0.01 * \exp((0.955/0.0702)(1-\exp(-0.0702GA)))$	5
$Vplacenta = 0 - 0.716GA + 0.9149GA^2 - 0.0122GA^3$	5
$Vamniotic\ fluid = 0 + 1.9648GA - 1.2056GA^2 + 0.2064GA^3 - 0.0061GA^4 + 0.00005GA^5$	5
$Vfat = 0.1305GA + 0.0008GA^2$	5
$CYP3A4 = 1 - 0.0016GA + 0.0019GA^2 - 0.00003GA^3$	9
$CYP2D6 = 1 + 0.022695GA - 0.000348GA^2$	5
$Fup = 1/(1+((1/Fu-1)/45.8)*(45.8 – 0.1775GA – 0.0033GA^2))$	5
CO: cardiac output; Fu: Fraction drug unbound in nonpregnancy; Fup: Fraction drug unbound in pregnancy; GA: gestational age; Q: blood flow; QGT: blood flow to gut; QLV: blood flow to liver; BW: body weight; V: volume	

Supplementary Table 5. Differential Equations Used in the Model

dxdt_LUMEN = - LUMEN*Ka;<br><br>dxdt_GUT = (Qgut*Cplasma + LUMEN*Ka) - Qgut*(Cgut/Kpgut) + (EHR*(((Cliver/Kpliver)*Fu)*Vmaxbile)/(Kmbile+(Cliver/Kpliver)*Fu));<br><br>dxdt_PLASMA = ((Qliver + Qgut) * ((Cliver) / Kpliver) + (Qslowperf*(Cslowperf/Kpslowperf)) + (Qrichperf * (Crichperf/Kprichperf))) - Cplasma * (Qliver + Qgut + Qrichperf + Qslowperf);<br><br>dxdt_LIVER = ((Qliver * Cplasma + Qgut * Cgut/Kpgut) - ((Cliver/Kpliver)*Fu)*CLhint - ((Qliver + Qgut) * (Cliver)/ Kpliver)) - (EHR*(((Cliver/Kpliver)*Fu)*Vmaxbile)/(Kmbile+(Cliver/Kpliver)*Fu));<br><br>dxdt_SLOWPERF = Qslowperf*(Cplasma - Cslowperf/Kpslowperf);<br><br>dxdt_RICHPERF = Qrichperf * (Cplasma - Crichperf/Kprichperf);
LUMEN: dosing compartment; Ka: Absorption constant; Q: Blood flow; C: Concentration; Kp: Tissue:plasma partition coefficient; Fu: Fraction of unbound drug; Slowperf: Slowly perfused tissue; Richperf: Richly perfused tissue; Vmaxbile: Maximal rate of transport in enterohepatic recirculation; Kmbile: Substrate concentration at which rate is half maximal in enterohepatic recirculation

References:

1. Obach, R. S., Cox, L. M. & Tremaine, L. M. Sertraline is metabolized by multiple cytochrome P450 enzymes, monoamine oxidases, and glucuronyl transferases in human: an in vitro study. *Drug Metab. Dispos.* **33**, 262–270 (2005).

2. Ronfeld, R. A., Tremaine, L. M. & Wilner, K. D. Pharmacokinetics of sertraline and its N-demethyl metabolite in elderly and young male and female volunteers. *Clin. Pharmacokinet.* **32**(Suppl 1), 22–30 (1997).

3. Wishart, F. Y. et al. DrugBank 5.0: a major update to the DrugBank database for 2018, *Nucleic Acids Res.* https://doi.org/10.1093/nar/gkx1037 (2017).

4. Poulin, P. & Haddad, S. Advancing prediction of tissue distribution and volume of distribution of highly lipophilic compounds from a simplified tissue-composition-based model as a mechanistic animal alternative method. *J. Pharm. Sci.* **101**, 2250–2261 (2012).

5. Abduljalil, K., Furness, P., Johnson, T. N., Rostami-Hodjegan, A. & Soltani, H. Anatomical, physiological and metabolic changes with gestational age during normal pregnancy: a database for parameters required in physiologically based pharmacokinetic modelling. *Clin. Pharmacokinet.* **51**, 365–396 (2012).

6. ICRP. Basic Anatomical and Physiological Data for Use in Radiological Protection Reference Values. ICRP Publication 89. *Ann. ICRP* **32**, 3-4 (2002).

7. Brown, R. P., Delp, M. D., Lindstedt, S. L., Rhomberg, L. R. & Beliles, R. P. Physiological parameter values for physiologically based pharmacokinetic models. *Toxicol. Ind. Health* **13**, 407-484 (1997).

8. Kapraun, D. F., Wambaugh, J. F., Setzer, R. W. & Judson, R. S. Empirical models for anatomical and physiological changes in a human mother and fetus during pregnancy and gestation. *PLoS ONE* **14**, e0215906 (2019).

9. Ke, A. B. & Milad, M. A. Evaluation of maternal drug exposure following the administration of antenatal corticosteroids during late pregnancy using physiologically-based pharmacokinetic modeling. *Clin. Pharmacol. Ther.* **106**, 164–173 (2019).
